# {*N*′-[(*E*)-1-(5-Bromo-2-oxidophen­yl)ethyl­idene]-4-hy­droxy­benzohydrazidato}pyridine­copper(II)

**DOI:** 10.1107/S1600536811011974

**Published:** 2011-04-07

**Authors:** Jian-Min Zhang, Liang Wang, Juan Liu, Yong-Chao Li, Hong-Ji Li

**Affiliations:** a College of Environment and Chemical Engineering, Xi’an Polytechnic University, 710048 Xi’an, Shaanxi, People’s Republic of China

## Abstract

In the title complex, [Cu(C_15_H_11_BrN_2_O_3_)(C_5_H_5_N)], the central Cu^II^ atom is in a square-planar CuN_2_O_2_ coordination environment formed by the tridentate hydrazone and the monodentate pyridine ligands with N atoms in a *trans*-arrangement about the Cu^II^ atom.

## Related literature

For the coordination properties of aroylhydrazones, see: Ali *et al.* (2004 ▶); Zheng *et al.* (2008 ▶) and for their biological activity, see: Carcelli *et al.* (1995 ▶).
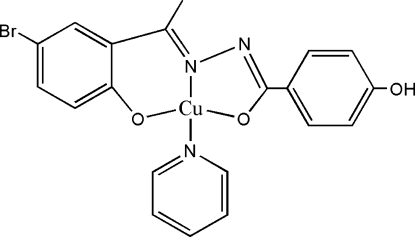


## Experimental

### 

#### Crystal data

[Cu(C_15_H_11_BrN_2_O_3_)(C_5_H_5_N)]
*M* *_r_* = 489.81
Monoclinic, 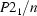

*a* = 12.514 (3) Å
*b* = 7.6539 (15) Å
*c* = 19.467 (4) Å
β = 93.276 (3)°
*V* = 1861.6 (6) Å^3^
*Z* = 4
Mo *K*α radiation
μ = 3.35 mm^−1^
*T* = 298 K
0.21 × 0.14 × 0.11 mm

#### Data collection

Bruker SMART CCD area-detector diffractometer
Absorption correction: multi-scan (*SADABS*; Sheldrick, 1996 ▶) *T*_min_ = 0.540, *T*_max_ = 0.710
9243 measured reflections
3293 independent reflections
2742 reflections with *I* > 2σ(*I*)
*R*_int_ = 0.041

#### Refinement

*R*[*F* ^2^ > 2σ(*F* ^2^)] = 0.040
*wR*(*F* ^2^) = 0.103
*S* = 1.02
3293 reflections
254 parameters
H-atom parameters constrained
Δρ_max_ = 0.53 e Å^−3^
Δρ_min_ = −0.89 e Å^−3^

### 

Data collection: *SMART* (Bruker, 1996 ▶); cell refinement: *SAINT* (Bruker, 1996 ▶); data reduction: *SAINT*; program(s) used to solve structure: *SHELXS97* (Sheldrick, 2008 ▶); program(s) used to refine structure: *SHELXL97* (Sheldrick, 2008 ▶); molecular graphics: *SHELXTL* (Sheldrick, 2008 ▶); software used to prepare material for publication: *SHELXTL*.

## Supplementary Material

Crystal structure: contains datablocks I, global. DOI: 10.1107/S1600536811011974/gk2354sup1.cif

Structure factors: contains datablocks I. DOI: 10.1107/S1600536811011974/gk2354Isup2.hkl

Additional supplementary materials:  crystallographic information; 3D view; checkCIF report

## Figures and Tables

**Fig. 1.**
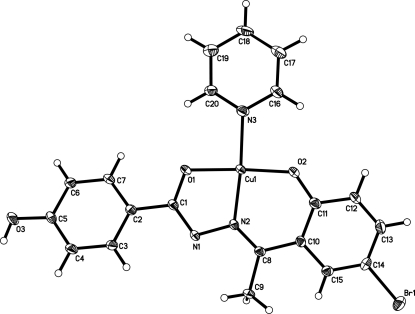
The molecular structure of the title compound with displacement ellipsoids drawn at the 30% probability level. H atoms are presented as a small spheres of arbitrary radius.

## supplementary crystallographic information

### Comment

The chemistry of aroylhydrazones continues to attract much attention due to their coordination ability towards metal ions (Zheng *et al.*, 2008; Ali *et al.*, 2004) and their biological activity (Carcelli *et al.*, 1995). As an extension of work on the structural characterization of aroylhydrazone derivatives, the title compound, C_20_H_16_BrCuN_3_O_3_, was synthesized and its crystal structure is reported here (Fig.1).

### Experimental

Ethyl 4-hydroxybenzoate (8.31 g, 0.05 mol) was dissolved in ethanol (30 ml) at room temperature and heated at 363 K, followed by the addition of hydrazine hydrate (3.25 g, 0.065 mol). Subsequently, the mixture was refluxed for 8 h, and then cooled to room temperature. The crystals were precipitated and collected by filtration. The product was recrystallized from ethanol and dried under reduced pressure to give (4-hydroxybenzoyl)hydrazine. (2-Hydroxybenzoyl) hydrazine (3.50 g, 0.023 mol) was dissolved in ethanol (30 ml) at room temperature and heated at 363 K, followed by the addition of 5-bromo-2-hydroxyphenyl ethyl ketone (4.95 g, 0.023 mol). Subsequently, the mixture was refluxed for 8 h, and then cooled to room temperature. The crystals were precipitated and collected by filtration. The product was recrystallized from ethanol and dried under reduced pressure to give *N*'-[( *E*)-(5-bromo-2-hydroxyphenyl)-ethylidene]-4-hydroxybenzohydrazide.

A methanol solution (10 ml) of *N*'-[(*E*)-(5-bromo-2-hydroxyphenyl) ethylidene]-4-hydroxybenzohydrazide (0.25 mmol, 0.087 g) was mixed with a DMF solution (5 ml) of CuCl_2_.2H_2_O (0.25 mmol, 0.043 g). The mixture was stirred at 298 K for 2 h. and then filtered. A blue precipitate was produced after about 15 days. A pyridine amount (5 ml) was used to dissolve the precipitate at 330 K. Blue block-shaped crystals of the title complex were obtained after one month (yield 30%).

### Refinement

All H atoms were positioned geometrically and treated as riding on their parent atoms,with *C*—*H*(methyl) = 0.96 Å, C—H(aromatic) = 0.93 Å, O—H = 0.82 Å and with *U*_iso_(H) =1.5*U*_eq_(C_methyl_,*O*) and 1.2*U*_eq_(C_aromatic_).

### Crystal data

**Table:**

[Cu(C_15_H_11_BrN_2_O_3_)(C_5_H_5_N)]	*F*(000) = 980
*M**_r_* = 489.81	*D*_x_ = 1.748 Mg m^−^^3^
Monoclinic, *P*2_1_/*n*	Mo *K*α radiation, λ = 0.71073 Å
Hall symbol: -P 2yn	Cell parameters from 4160 reflections
*a* = 12.514 (3) Å	θ = 2.9–27.6°
*b* = 7.6539 (15) Å	µ = 3.35 mm^−^^1^
*c* = 19.467 (4) Å	*T* = 298 K
β = 93.276 (3)°	Block, blue
*V* = 1861.6 (6) Å^3^	0.21 × 0.14 × 0.11 mm
*Z* = 4	

### Data collection

**Table:**

Bruker SMART CCD area-detector diffractometer	3293 independent reflections
Radiation source: fine-focus sealed tube	2742 reflections with *I* > 2σ(*I*)
graphite	*R*_int_ = 0.041
phi and ω scans	θ_max_ = 25.0°, θ_min_ = 1.9°
Absorption correction: multi-scan (*SADABS*; Sheldrick, 1996)	*h* = −14→14
*T*_min_ = 0.540, *T*_max_ = 0.710	*k* = −8→9
9243 measured reflections	*l* = −19→23

### Refinement

**Table:**

Refinement on *F*^2^	Secondary atom site location: difference Fourier map
Least-squares matrix: full	Hydrogen site location: inferred from neighbouring sites
*R*[*F*^2^ > 2σ(*F*^2^)] = 0.040	H-atom parameters constrained
*wR*(*F*^2^) = 0.103	*w* = 1/[σ^2^(*F*_o_^2^) + (0.064*P*)^2^] where *P* = (*F*_o_^2^ + 2*F*_c_^2^)/3
*S* = 1.02	(Δ/σ)_max_ = 0.001
3293 reflections	Δρ_max_ = 0.53 e Å^−^^3^
254 parameters	Δρ_min_ = −0.89 e Å^−^^3^
0 restraints	Extinction correction: *SHELXL*
Primary atom site location: structure-invariant direct methods	Extinction coefficient: 0.0113 (15)

### Special details

**Table:**

Geometry. All e.s.d.'s (except the e.s.d. in the dihedral angle between two l.s. planes) are estimated using the full covariance matrix. The cell e.s.d.'s are taken into account individually in the estimation of e.s.d.'s in distances, angles and torsion angles; correlations between e.s.d.'s in cell parameters are only used when they are defined by crystal symmetry. An approximate (isotropic) treatment of cell e.s.d.'s is used for estimating e.s.d.'s involving l.s. planes.
Refinement. Refinement of *F*^2^ against ALL reflections. The weighted *R*-factor *wR* and goodness of fit *S* are based on *F*^2^, conventional *R*-factors *R* are based on *F*, with *F* set to zero for negative *F*^2^. The threshold expression of *F*^2^ > σ(*F*^2^) is used only for calculating *R*-factors(gt) *etc*. and is not relevant to the choice of reflections for refinement. *R*-factors based on *F*^2^ are statistically about twice as large as those based on *F*, and *R*- factors based on ALL data will be even larger.

### Fractional atomic coordinates and isotropic or equivalent isotropic displacement parameters (Å^2^)

**Table:**

	*x*	*y*	*z*	*U*_iso_*/*U*_eq_	
Cu1	0.42814 (3)	0.76855 (5)	0.51714 (2)	0.02479 (15)	
Br1	0.72536 (3)	0.37728 (5)	0.24220 (2)	0.04230 (16)	
O1	0.45528 (17)	0.8886 (3)	0.60153 (13)	0.0317 (6)	
O2	0.41045 (18)	0.6643 (3)	0.43203 (13)	0.0356 (6)	
O3	0.6722 (2)	1.2098 (4)	0.86987 (14)	0.0530 (8)	
H3	0.7331	1.2496	0.8719	0.079*	
N1	0.6306 (2)	0.8483 (3)	0.57583 (14)	0.0255 (6)	
N2	0.5824 (2)	0.7708 (3)	0.51648 (14)	0.0231 (6)	
N3	0.2686 (2)	0.7744 (4)	0.52600 (15)	0.0297 (7)	
C1	0.5564 (2)	0.9039 (4)	0.61532 (17)	0.0243 (7)	
C2	0.5902 (2)	0.9870 (4)	0.68103 (16)	0.0245 (7)	
C3	0.6889 (2)	1.0679 (4)	0.69190 (17)	0.0264 (7)	
H3A	0.7361	1.0699	0.6567	0.032*	
C4	0.7182 (3)	1.1457 (4)	0.75435 (18)	0.0290 (8)	
H4	0.7841	1.2013	0.7606	0.035*	
C5	0.6498 (3)	1.1405 (4)	0.80707 (19)	0.0324 (8)	
C6	0.5506 (3)	1.0600 (5)	0.79668 (19)	0.0386 (9)	
H6	0.5044	1.0549	0.8324	0.046*	
C7	0.5207 (3)	0.9881 (5)	0.73399 (18)	0.0337 (8)	
H7	0.4529	0.9395	0.7269	0.040*	
C8	0.6428 (2)	0.7196 (4)	0.46905 (17)	0.0227 (7)	
C9	0.7609 (3)	0.7525 (5)	0.4745 (2)	0.0325 (8)	
H9A	0.7758	0.8521	0.5035	0.049*	
H9B	0.7857	0.7746	0.4296	0.049*	
H9C	0.7970	0.6519	0.4941	0.049*	
C10	0.5969 (3)	0.6315 (4)	0.40775 (18)	0.0231 (7)	
C11	0.4854 (3)	0.6139 (4)	0.39242 (19)	0.0280 (8)	
C12	0.4511 (3)	0.5377 (5)	0.33003 (18)	0.0326 (8)	
H12	0.3780	0.5311	0.3189	0.039*	
C13	0.5190 (3)	0.4728 (5)	0.28489 (18)	0.0358 (9)	
H13	0.4930	0.4229	0.2437	0.043*	
C14	0.6286 (3)	0.4821 (4)	0.30120 (17)	0.0288 (7)	
C15	0.6662 (3)	0.5614 (4)	0.36049 (17)	0.0259 (7)	
H15	0.7398	0.5693	0.3699	0.031*	
C16	0.2007 (3)	0.7253 (5)	0.4739 (2)	0.0430 (10)	
H16	0.2280	0.6883	0.4329	0.052*	
C17	0.0928 (3)	0.7282 (7)	0.4793 (2)	0.0594 (13)	
H17	0.0475	0.6944	0.4421	0.071*	
C18	0.0502 (3)	0.7811 (6)	0.5396 (3)	0.0556 (12)	
H18	−0.0234	0.7846	0.5437	0.067*	
C19	0.1188 (3)	0.8281 (6)	0.5930 (2)	0.0472 (10)	
H19	0.0931	0.8630	0.6347	0.057*	
C20	0.2280 (3)	0.8229 (5)	0.58405 (19)	0.0338 (8)	
H20	0.2748	0.8551	0.6207	0.041*	

### Atomic displacement parameters (Å^2^)

**Table:**

	*U*^11^	*U*^22^	*U*^33^	*U*^12^	*U*^13^	*U*^23^
Cu1	0.0153 (2)	0.0365 (2)	0.0219 (3)	0.00162 (15)	−0.00401 (17)	−0.00250 (17)
Br1	0.0465 (3)	0.0481 (3)	0.0335 (3)	0.00384 (16)	0.01232 (19)	−0.00779 (17)
O1	0.0164 (12)	0.0502 (15)	0.0277 (14)	0.0023 (10)	−0.0067 (10)	−0.0094 (11)
O2	0.0192 (12)	0.0575 (15)	0.0292 (15)	0.0015 (11)	−0.0057 (11)	−0.0127 (12)
O3	0.0337 (15)	0.095 (2)	0.0296 (16)	−0.0228 (15)	−0.0001 (12)	−0.0242 (15)
N1	0.0195 (14)	0.0354 (15)	0.0210 (16)	−0.0035 (11)	−0.0039 (12)	−0.0041 (12)
N2	0.0196 (14)	0.0312 (14)	0.0178 (15)	0.0001 (10)	−0.0038 (12)	0.0006 (11)
N3	0.0224 (15)	0.0388 (16)	0.0276 (17)	0.0002 (12)	−0.0012 (13)	0.0051 (13)
C1	0.0197 (17)	0.0311 (17)	0.0214 (18)	0.0005 (13)	−0.0045 (14)	0.0003 (14)
C2	0.0193 (16)	0.0308 (17)	0.0230 (18)	0.0027 (13)	−0.0032 (14)	0.0006 (14)
C3	0.0188 (16)	0.0345 (17)	0.0257 (19)	−0.0001 (13)	0.0008 (14)	−0.0004 (15)
C4	0.0176 (17)	0.0382 (19)	0.030 (2)	−0.0009 (13)	−0.0040 (15)	−0.0001 (15)
C5	0.0245 (18)	0.048 (2)	0.023 (2)	−0.0023 (15)	−0.0085 (15)	−0.0075 (16)
C6	0.0228 (18)	0.068 (2)	0.025 (2)	−0.0051 (17)	0.0032 (15)	−0.0115 (19)
C7	0.0183 (17)	0.050 (2)	0.032 (2)	−0.0045 (15)	−0.0039 (15)	−0.0083 (17)
C8	0.0169 (16)	0.0286 (16)	0.0222 (18)	0.0019 (12)	−0.0025 (14)	0.0034 (14)
C9	0.0147 (17)	0.052 (2)	0.030 (2)	−0.0027 (14)	−0.0018 (15)	−0.0046 (16)
C10	0.0234 (17)	0.0261 (16)	0.0191 (17)	0.0019 (12)	−0.0034 (14)	0.0020 (13)
C11	0.0223 (17)	0.0307 (18)	0.030 (2)	0.0036 (13)	−0.0042 (15)	0.0006 (15)
C12	0.0229 (18)	0.045 (2)	0.029 (2)	0.0006 (15)	−0.0080 (15)	−0.0061 (16)
C13	0.040 (2)	0.039 (2)	0.028 (2)	−0.0002 (16)	−0.0065 (17)	−0.0088 (16)
C14	0.036 (2)	0.0299 (17)	0.0218 (18)	0.0015 (14)	0.0086 (15)	−0.0006 (15)
C15	0.0218 (17)	0.0303 (17)	0.0256 (18)	0.0016 (13)	0.0005 (14)	0.0051 (15)
C16	0.027 (2)	0.070 (3)	0.031 (2)	−0.0003 (18)	−0.0073 (17)	−0.0030 (19)
C17	0.026 (2)	0.103 (4)	0.048 (3)	−0.007 (2)	−0.008 (2)	−0.007 (3)
C18	0.017 (2)	0.089 (3)	0.061 (3)	−0.0056 (19)	0.001 (2)	−0.002 (3)
C19	0.031 (2)	0.065 (3)	0.047 (3)	0.0008 (18)	0.0078 (19)	−0.003 (2)
C20	0.0223 (19)	0.049 (2)	0.030 (2)	−0.0006 (15)	0.0041 (16)	0.0003 (17)

### Geometric parameters (Å, °)

**Table:**

Cu1—O2	1.841 (2)	C7—H7	0.9300
Cu1—O1	1.896 (2)	C8—C10	1.460 (4)
Cu1—N2	1.931 (3)	C8—C9	1.497 (4)
Cu1—N3	2.015 (3)	C9—H9A	0.9600
Br1—C14	1.894 (3)	C9—H9B	0.9600
O1—C1	1.284 (4)	C9—H9C	0.9600
O2—C11	1.306 (4)	C10—C15	1.407 (5)
O3—C5	1.347 (4)	C10—C11	1.416 (5)
O3—H3	0.8200	C11—C12	1.393 (5)
N1—C1	1.310 (4)	C12—C13	1.351 (5)
N1—N2	1.404 (4)	C12—H12	0.9300
N2—C8	1.287 (4)	C13—C14	1.392 (5)
N3—C20	1.318 (5)	C13—H13	0.9300
N3—C16	1.339 (5)	C14—C15	1.364 (5)
C1—C2	1.469 (4)	C15—H15	0.9300
C2—C7	1.386 (5)	C16—C17	1.361 (6)
C2—C3	1.388 (4)	C16—H16	0.9300
C3—C4	1.384 (5)	C17—C18	1.377 (6)
C3—H3A	0.9300	C17—H17	0.9300
C4—C5	1.374 (5)	C18—C19	1.358 (6)
C4—H4	0.9300	C18—H18	0.9300
C5—C6	1.391 (5)	C19—C20	1.388 (5)
C6—C7	1.371 (5)	C19—H19	0.9300
C6—H6	0.9300	C20—H20	0.9300
			
O2—Cu1—O1	175.06 (11)	C8—C9—H9A	109.5
O2—Cu1—N2	93.83 (11)	C8—C9—H9B	109.5
O1—Cu1—N2	82.63 (10)	H9A—C9—H9B	109.5
O2—Cu1—N3	91.02 (11)	C8—C9—H9C	109.5
O1—Cu1—N3	92.55 (11)	H9A—C9—H9C	109.5
N2—Cu1—N3	175.14 (11)	H9B—C9—H9C	109.5
C1—O1—Cu1	110.6 (2)	C15—C10—C11	117.6 (3)
C11—O2—Cu1	127.3 (2)	C15—C10—C8	118.8 (3)
C5—O3—H3	109.5	C11—C10—C8	123.6 (3)
C1—N1—N2	109.5 (3)	O2—C11—C12	116.3 (3)
C8—N2—N1	118.5 (3)	O2—C11—C10	125.4 (3)
C8—N2—Cu1	129.1 (2)	C12—C11—C10	118.3 (3)
N1—N2—Cu1	112.4 (2)	C13—C12—C11	123.2 (3)
C20—N3—C16	118.0 (3)	C13—C12—H12	118.4
C20—N3—Cu1	120.8 (2)	C11—C12—H12	118.4
C16—N3—Cu1	121.2 (3)	C12—C13—C14	118.7 (3)
O1—C1—N1	124.8 (3)	C12—C13—H13	120.6
O1—C1—C2	116.9 (3)	C14—C13—H13	120.6
N1—C1—C2	118.3 (3)	C15—C14—C13	120.2 (3)
C7—C2—C3	118.3 (3)	C15—C14—Br1	120.0 (3)
C7—C2—C1	119.3 (3)	C13—C14—Br1	119.7 (3)
C3—C2—C1	122.4 (3)	C14—C15—C10	121.8 (3)
C4—C3—C2	121.0 (3)	C14—C15—H15	119.1
C4—C3—H3A	119.5	C10—C15—H15	119.1
C2—C3—H3A	119.5	N3—C16—C17	121.7 (4)
C5—C4—C3	119.9 (3)	N3—C16—H16	119.1
C5—C4—H4	120.0	C17—C16—H16	119.1
C3—C4—H4	120.0	C16—C17—C18	120.3 (4)
O3—C5—C4	124.1 (3)	C16—C17—H17	119.8
O3—C5—C6	116.3 (3)	C18—C17—H17	119.8
C4—C5—C6	119.6 (3)	C19—C18—C17	118.2 (4)
C7—C6—C5	120.2 (3)	C19—C18—H18	120.9
C7—C6—H6	119.9	C17—C18—H18	120.9
C5—C6—H6	119.9	C18—C19—C20	118.7 (4)
C6—C7—C2	120.9 (3)	C18—C19—H19	120.6
C6—C7—H7	119.5	C20—C19—H19	120.6
C2—C7—H7	119.5	N3—C20—C19	123.1 (4)
N2—C8—C10	120.5 (3)	N3—C20—H20	118.5
N2—C8—C9	121.0 (3)	C19—C20—H20	118.5
C10—C8—C9	118.5 (3)		
			
N2—Cu1—O1—C1	−2.0 (2)	N1—N2—C8—C10	177.3 (3)
N3—Cu1—O1—C1	177.3 (2)	Cu1—N2—C8—C10	−6.2 (4)
N2—Cu1—O2—C11	1.3 (3)	N1—N2—C8—C9	−4.1 (4)
N3—Cu1—O2—C11	−178.3 (3)	Cu1—N2—C8—C9	172.3 (2)
C1—N1—N2—C8	175.0 (3)	N2—C8—C10—C15	−173.5 (3)
C1—N1—N2—Cu1	−2.0 (3)	C9—C8—C10—C15	8.0 (4)
O2—Cu1—N2—C8	2.2 (3)	N2—C8—C10—C11	7.2 (5)
O1—Cu1—N2—C8	−174.4 (3)	C9—C8—C10—C11	−171.3 (3)
O2—Cu1—N2—N1	178.77 (19)	Cu1—O2—C11—C12	179.9 (2)
O1—Cu1—N2—N1	2.24 (19)	Cu1—O2—C11—C10	−0.4 (5)
O2—Cu1—N3—C20	172.7 (3)	C15—C10—C11—O2	176.8 (3)
O1—Cu1—N3—C20	−10.8 (3)	C8—C10—C11—O2	−4.0 (5)
O2—Cu1—N3—C16	−5.7 (3)	C15—C10—C11—C12	−3.6 (4)
O1—Cu1—N3—C16	170.9 (3)	C8—C10—C11—C12	175.7 (3)
Cu1—O1—C1—N1	1.6 (4)	O2—C11—C12—C13	−177.3 (3)
Cu1—O1—C1—C2	−177.1 (2)	C10—C11—C12—C13	3.1 (5)
N2—N1—C1—O1	0.3 (4)	C11—C12—C13—C14	0.0 (6)
N2—N1—C1—C2	179.0 (3)	C12—C13—C14—C15	−2.5 (5)
O1—C1—C2—C7	22.6 (4)	C12—C13—C14—Br1	175.4 (3)
N1—C1—C2—C7	−156.1 (3)	C13—C14—C15—C10	1.8 (5)
O1—C1—C2—C3	−156.1 (3)	Br1—C14—C15—C10	−176.1 (2)
N1—C1—C2—C3	25.1 (5)	C11—C10—C15—C14	1.3 (5)
C7—C2—C3—C4	0.9 (5)	C8—C10—C15—C14	−178.0 (3)
C1—C2—C3—C4	179.7 (3)	C20—N3—C16—C17	1.4 (6)
C2—C3—C4—C5	1.2 (5)	Cu1—N3—C16—C17	179.8 (3)
C3—C4—C5—O3	178.8 (3)	N3—C16—C17—C18	−0.5 (7)
C3—C4—C5—C6	−1.3 (5)	C16—C17—C18—C19	−0.6 (8)
O3—C5—C6—C7	179.1 (4)	C17—C18—C19—C20	0.8 (7)
C4—C5—C6—C7	−0.8 (6)	C16—N3—C20—C19	−1.1 (6)
C5—C6—C7—C2	3.0 (6)	Cu1—N3—C20—C19	−179.6 (3)
C3—C2—C7—C6	−3.1 (5)	C18—C19—C20—N3	0.0 (6)
C1—C2—C7—C6	178.1 (3)		

### Hydrogen-bond geometry (Å, °)

**Table:**

*D*—H···*A*	*D*—H	H···*A*	*D*···*A*	*D*—H···*A*
O3—H3···N1^i^	0.82	2.08	2.834 (4)	153.

Symmetry codes: (i) −*x*+3/2, *y*+1/2, −*z*+3/2.
